# How Classmates’ Gender Stereotypes Affect Students’ Math Self-Concepts: A Multilevel Analysis

**DOI:** 10.3389/fpsyg.2021.599199

**Published:** 2021-05-12

**Authors:** Fabian Wolff

**Affiliations:** Institute of Psychology, University of Koblenz-Landau, Koblenz, Germany

**Keywords:** academic self-concept, beliefs, classmates, contextual effects, gender stereotypes, math self-concept, peers

## Abstract

The present research is the first to examine how students’ individual and their classmates’ math-related gender stereotypes, endorsing that math would be a typically male domain, relate to students’ math self-concepts. To this end, data of *N* = 1,424 secondary school students from Germany were analyzed using multilevel analyses. As expected, strong individual beliefs in the math-related gender stereotype were related to lower math self-concepts for girls, but to higher math self-concepts for boys. Moreover, classmates’ shared beliefs in this stereotype showed a negative relation to girls’ self-concepts, whereas no significant relation between classmates’ shared beliefs and boys’ self-concepts was found. These relations also persisted after controlling for students’ math grades and age. In sum, the results demonstrated that gender stereotypes shared by students’ classmates can have a substantial impact on students’ math self-concepts, beyond their individual gender stereotypes. This finding emphasizes the significance of classmates as important socializing peers in the process of students’ self-concept formation.

## Introduction

Although gender differences in math performance have largely vanished today, female students still tend to consider themselves as being less competent in math than their male counterparts (e.g., Hyde et al., 2008; Niepel et al., 2020). These gender differences in students’ math self-concepts remain a cause for concern—not only because subject-specific self-concepts strongly relate to students’ attitudes and affects toward school subjects (e.g., Goetz et al., 2008; Schurtz et al., 2014), but also because students include their self-concepts in their career choices (e.g., Parker et al., 2012, 2014). For that reason, the lower math self-concepts of females might hamper current political efforts to reduce the underrepresentation of women in fields related to math, science, technology, and engineering [Niepel et al., 2020; Organisation for Economic Co-operation and Development (OECD), 2020].

In the last decades, gender differences in students’ math self-concepts have been examined in plenty of studies (e.g., Eccles-Parsons et al., 1983; Skaalvik and Skaalvik, 2004; Preckel et al., 2008). Among these, some studies have addressed the question of how gender differences in students’ math self-concepts depend on gender stereotypes. Interestingly enough, these studies found that the gender differences in students’ math self-concepts are larger, the more students believe that math would be a subject for boys rather than for girls (e.g., Nosek et al., 2002; Steffens and Jelenec, 2011; Passolunghi et al., 2014). However, all of these studies only considered the relations between students’ math self-concepts and their math-related gender stereotypes at the individual level, while neglecting the potential effects of math-related gender stereotypes shared by students’ classmates. Given prior findings on the impact of significant adults’ (teachers’ and parents’) math-related gender stereotypes on students’ attitudes toward math (e.g., Gunderson et al., 2012), as well as the role of classmates as important socializing peers (e.g., Berndt and Murphy, 2002), it would seem reasonable to assume that classmates’ math-related gender stereotypes affect students’ math self-concepts beyond their individual stereotypes. Moreover, Muntoni et al. (2021) provided empirical evidence that students’ self-concepts are affected by gender stereotypes shared in the classroom. In this study, the authors found that boys’ reading self-concepts were lower, the more their classmates believed in the stereotype that reading would be a typically female domain. However, although most research on the relations between subject-specific gender stereotypes and students’ self-concepts has focused on the math domain, no empirical study has so far examined whether Muntoni et al.’s (2021) findings would also apply to the math domain (i.e., whether girls show lower math self-concepts, the more their classmates believe in the stereotype favoring boys in math).

The present research addresses this research gap. To this end, I will analyze data collected in a large sample of secondary school students from Germany who reported both their math self-concepts and their math-related gender stereotypes. For the first time, I will examine whether and how girls’ and boys’ math self-concepts are affected by math-related gender stereotypes held by themselves and shared by their classmates. Thus, this study will make an important contribution to the literature by significantly enhancing our knowledge of the emergence of gender differences in students’ math self-concepts. Beyond that, it is the first study to test whether Muntoni et al.’s (2021) findings—which have received considerable attention not only in the scientific community [e.g., Gesellschaft für Empirische Bildungsforschung (GEBF), 2019] but also in the media (e.g., Deutsches Schulportal, 2020; Society for Research in Child Development, 2020)—will generalize to another domain. This research will therefore provide us with valuable information to assess whether Muntoni et al. (2021) have discovered a psychological mechanism that operates across domains, or whether the relations shown in their study may be more specific to the domain of reading.

### Impact of Students’ Own Gender Stereotypes on Their Math Self-Concepts

Stereotypes can be defined as shared beliefs about traits that are characteristic of members of a certain social category (e.g., Greenwald and Banaji, 1995). Although they may facilitate human behavior and decision-making in complex environments, they often come along with negative effects for the stereotyped group (e.g., Steele and Aronson, 1995; Schmader et al., 2004; Ihme and Möller, 2015). Concerning math, prior studies on gender stereotypes have repeatedly found support for the prevalence of the stereotype endorsing that math would be a typically male domain (e.g., Nosek et al., 2002; Steffens et al., 2010; Cvencek et al., 2011; Steffens and Jelenec, 2011; but see Plante et al., 2009). This finding is somewhat paradoxical in that girls do not (any longer) perform significantly worse than boys in math [e.g., Hyde et al., 2008; Else-Quest et al., 2010; Lindberg et al., 2010; Niepel et al., 2020; Organisation for Economic Co-operation and Development (OECD), 2020] and even receive slightly better math grades (Voyer and Voyer, 2014). Yet, the prevailing view that males would be more competent in math may be a key reason as to why girls on average report lower math self-concepts than boys (e.g., Skaalvik and Skaalvik, 2004; Niepel et al., 2020) and thus develop less positive attitudes and affects toward math (e.g., Else-Quest et al., 2010; Goetz et al., 2013; Schurtz et al., 2014; Arens et al., 2017).

A prominent theory predicting these relations is Eccles’ expectancy-value theory—a comprehensive model for the explanation of achievement-related choices and behaviors (EVT; Eccles-Parsons et al., 1983; Wigfield and Eccles, 2000; Eccles, 2009). EVT was originally developed to help explain gender differences in the likelihood of studying math and science. Among others, it assumes that stereotypes shared within one culture, such as beliefs about gender roles, affect students’ self-concepts, and consequently their subject-specific task values and other achievement-related outcomes. However, EVT also assumes that students’ perceptions of these stereotypes significantly account for the stereotypes’ impact on students’ self-concepts. In line with this assumption, several studies have shown that the influence of the math-related gender stereotype favoring boys in math on students’ math self-concepts depends on students’ beliefs in this stereotype (e.g., Nosek et al., 2002; Kurtz-Costes et al., 2008; Evans et al., 2011; Steffens and Jelenec, 2011; Passolunghi et al., 2014). More specifically, these studies revealed that girls tend to show lower math self-concepts, whereas boys typically have higher math self-concepts, the more they believe in this stereotype. Furthermore, some studies have shown that the effect of math-related gender stereotypes can even be reversed in favor of girls if students—against cultural conventions—are convinced that girls would be more competent in math than boys (e.g., Passolunghi et al., 2014).

To sum up, the stereotype endorsing that math would be a typically male domain does not affect each student’s math self-concept in the same way. Rather, girls’ and boys’ beliefs regarding the validity of this stereotype are central determinants for its influence on their math self-concepts. Based on these findings, the present study further examines how students’ beliefs in the gender stereotype favoring boys in math affect their math self-concepts by considering, for the first time, the potential influence of shared beliefs about this stereotype in students’ classrooms.

### Impact of Classmates’ Gender Stereotypes on Students’ Self-Concepts

Besides the impact of students’ individual gender stereotypes, previous research has found that students’ self-concepts are also affected by gender stereotypes held by significant others. These studies have dealt with the influence of parents’ and teachers’ beliefs (e.g., Jacobs and Eccles, 1992; Bouchey, 2004; Gunderson et al., 2012; Rowley et al., 2013; Muntoni and Retelsdorf, 2019). For example, Jacobs and Eccles (1992), analyzing longitudinal data, found that the math-related gender stereotypes of mothers influenced their perceptions of their children’s math abilities in such a way that mothers with a strong belief in the stereotype favoring boys in math enhanced their perceived abilities of their sons but devalued their perceived abilities of their daughters. In turn, these ability perceptions affected the children’s own math self-concepts, beyond students’ actual gender and their teachers’ beliefs about their abilities. These findings are also in accord with EVT, which emphasizes the role of socializers’ beliefs and predicts that these beliefs, such as those about gender stereotypes, affect students’ self-concepts even beyond students’ own beliefs about gender stereotypes.

Unlike the relatively high number of studies examining the impact of parents’ and teachers’ gender stereotypes on students’ math self-concepts, the impact of classmates’ gender stereotypes on students’ math self-concepts has not been examined so far. Nevertheless, such an influence seems plausible, given that peers also represent important socializers for students, especially during adolescence (e.g., Berndt, 1979; Hartup, 1996; Espelage et al., 2003; Brown, 2011). In particular, previous research has shown that peers can be of high importance for imparting behavior connoting appropriateness based on gender (e.g., Lamb et al., 1980; Zucker and Bradley, 1995; Bussey and Bandura, 1999; Ruble et al., 2007). Moreover, several studies have found that students’ individual beliefs and values can be significantly affected by those of their classmates (e.g., Ryan, 2001; Bouchey, 2004; Frenzel et al., 2010; Studsrød and Bru, 2011). For example, with regard to math, Bouchey (2004) showed in a cross-sectional study that classmates’ ratings of students’ math ability were positively related to students’ math self-concepts, after controlling for students’ math achievement. Besides, Frenzel et al. (2010) found in a longitudinal study that students’ math interest was higher, the more value their classmates expressed toward math. These authors also noted that classmates can be assumed to play an important role in the formation of students’ achievement-related beliefs and values, especially in Germany, as students in the German school system stay within the same group of students across most subjects within a school year.

More specific evidence for the assumption that students’ math self-concepts might be influenced by their classmates’ math-related gender stereotypes stems from two longitudinal studies, in which data collected in Germany were re-analyzed. In the one study, Salikutluk and Heyne (2017) investigated how classmates’ gender norms impact on students’ math achievement. They found that girls performed worse in classes where traditional gender norms were strongly present, whereas girls’ and boys’ math achievement did not differ in classes where traditional gender norms were low or absent. In the other study, Muntoni et al. (2021) examined the relations between reading-related gender stereotypes and reading self-concepts. As already stated in the Introduction, they found that boys developed lower reading self-concepts, the more their classmates believed in the stereotype favoring girls in reading, whereas classmates’ beliefs in this stereotype did not affect girls’ reading self-concepts. Similar findings also emerged for students’ reading self-efficacy, reading motivation, and reading achievement.

Taken together, the results of both studies suggest that high subject-specific gender stereotypes shared within the classroom might negatively impact on the stereotyped group. However, it should be noted that the gender norms examined by Salikutluk and Heyne (2017) did not refer to school subjects, but particularly addressed the question of whether men should use violence to defend their families. Thus, it is unclear whether Salikutluk and Heyne’s findings would be replicated if gender norms (or gender stereotypes) referred to math. In particular, it is worth noting that students’ individual gender norms in Salikutluk and Heyne’s study were negatively related to math achievement not only for girls but also for boys—presumably because a strong belief that men should use violence might go along with more antisocial behavior at school and thus with lower math achievement for boys. Moreover, it is questionable to what extent the effects of gender norms on students’ math achievement correspond to those on their math self-concept, given that the gender gap in math self-concept is significantly stronger than in math achievement (e.g., Niepel et al., 2020).

In contrast to Salikutluk and Heyne (2017), Muntoni et al. (2021) investigated the impact of classmates’ subject-specific gender stereotypes on students’ self-concepts more specifically. Notwithstanding this, it cannot be taken for granted that their results would generalize to the math domain. This is especially true because the stereotyped gender groups in the domains of reading and math differ from each other. For example, an important difference between stereotypes relating to boys’ reading competence versus girls’ math competence is that boys, in fact, perform worse in reading, whereas the gender difference in students’ math performance is at least very small [e.g., Organisation for Economic Co-operation and Development (OECD), 2020]. Thus, one could speculate that classmates’ subject-specific gender stereotypes affect boys’ reading self-concepts more strongly than girls’ math self-concepts because the stereotype favoring girls in reading is objectively more tenable. Aside from that, it is worth mentioning that the contextual effect on boys’ reading self-concepts found by Muntoni et al. (2021) strongly resulted from an absence of the effect of boys’ individual reading-related gender stereotypes on their reading self-concepts. However, concerning the math domain, such a finding would contradict the various findings on the impact of students’ individual beliefs in the stereotype favoring boys in math on their math self-concepts (e.g., Passolunghi et al., 2014). This also makes it necessary to investigate whether Muntoni et al.’s (2021) findings can be generalized to the domain of math.

To summarize, it seems likely that classmates’ math-related gender stereotypes affect students’ math self-concepts beyond their individual gender stereotypes. Nevertheless, this is an empirical question, which has not yet been addressed in any study. The present study aims to close this research gap.

## The Present Research

This research is the first to investigate the relations between students’ and their classmates’ math-related gender stereotypes and students’ math self-concepts. Its main purpose is to examine the influence of classmates’ gender stereotypes on students’ self-concepts. Thus, for the first time, it aims to test whether Muntoni et al.’s (2021) much-noticed findings concerning the relations between classmates’ reading-related gender stereotypes and students’ reading self-concepts would generalize to the domain of math. Moreover, this study seeks to dive further into the question as to what extent students’ individual beliefs concerning math-related gender stereotypes affect their math self-concepts by examining these relations, separately for girls and boys, after controlling for the relations between gender stereotypes and self-concepts at the class level.

Based on the theoretical reflections and empirical findings presented in the previous sections, I assumed to find a negative relation between girls’ individual beliefs in the gender stereotype favoring boys in math and their math self-concepts (*Hypothesis 1a*). On the contrary, I assumed to find a positive relation between boys’ individual beliefs in this stereotype and their math self-concepts (*Hypothesis 1b*). Furthermore, I expected that girls’ math self-concepts showed a negative relation to their classmates’ beliefs in the gender stereotype favoring boys in math (*Hypothesis 2*), given that previous research suggests that high subject-specific gender stereotypes shared within the classroom might negatively impact on the stereotyped group (Salikutluk and Heyne, 2017; Muntoni et al., 2021). That is, I assumed that classmates’ math-related gender stereotypes would show a negative relation to girls’ math self-concepts, after controlling for students’ individual differences in their math-related gender stereotypes. I left it as an open research question whether classmates’ beliefs in the gender stereotype favoring boys in math would also relate to boys’ math self-concepts. For example, whereas some studies have found that stereotypes questioning the ability of an outgroup can lead to performance boosts in the ingroup (“stereotype lift”; e.g., Walton and Cohen, 2003), other studies have found that they can decrease the performance in the ingroup (“choking under pressure”; e.g., Cheryan and Bodenhausen, 2000). Nevertheless, given that Muntoni et al. (2021) found no significant effect of classmates’ beliefs in the stereotype favoring girls in reading on girls’ reading self-concepts, I considered it as likely that boys’ math self-concepts would not significantly relate to their classmates’ beliefs in the stereotype favoring boys in math.

I tested my hypotheses in a large sample of secondary school students from Germany. To account for potential alternative explanations, I also tested the relations between students’ math-related gender stereotypes and math self-concepts after controlling for students’ math grades and age. I controlled for students’ math grades, as students form their subject-specific self-concepts especially based on their grades. In particular, previous research has shown that students’ math self-concepts show significantly stronger relations to their math grades than to their results from standardized math tests (e.g., Wolff et al., 2019b; Möller et al., 2020). I took into account students’ age since students’ math self-concepts usually decrease with increasing age (e.g., Wolff et al., 2020b; Orth et al., 2021). I expected to find support for all stated hypotheses even after controlling for the covariates.

## Materials and Methods

### Sample

The sample consisted of *N* = 1,424 students (age: *M* = 15.1, *SD* = 2.01; 56.2% female, 42.6% male, 1.2% not specified) between Grade 7 and Grade 13. These students attended 90 classes of 11 secondary schools in the German federal state of Schleswig-Holstein. The number of classes per school ranged from 2 to 14 with an average of about 8 classes per school. The sample stemmed from a larger project with the major aim to examine comparison effects in the process of students’ self-concept formation (Wolff et al., 2021). The schools were recruited by direct requests to individual school offices. Participating classes were selected by school administrators. In many cases, there was direct contact with specific teachers at the schools who offered to participate in the study, particularly with classes they taught themselves. The participation was voluntary and the informed consent of the parents was required if students were underage. A lack of informed parental consent was the main reason why students did not participate in the study. It mostly resulted from the fact that the students had not handed over the parent letter in advance of the study.

### Procedure

The data collection took place in the spring of 2018. The students answered paper-and-pencil questionnaires during regular school lessons. The constructs were measured in the following order: math self-concept, math-related gender stereotype, math grade, and demographics (age and gender). Approval for the whole procedure was obtained by the local ministry of education.

### Measures

#### Math Self-Concepts

Students’ math self-concepts were measured using six items: (1) “With a number of things in math, I immediately know: I will never understand this,” (2) “Although I try my best, math is hard for me,” (3) “I simply have no natural aptitude in math,” (4) “I would much rather do math if the subject were not so difficult,” (5) “Math does not come naturally to me,” (6) “It comes easily to me to understand tasks and solve problems in math.” These items had already been used successfully in contemporary self-concept research (e.g., Wolff et al., 2019a). The students responded to all items on a 6-point Likert scale ranging from 1 = *strongly disagree* to 6 = *strongly agree*. The negatively phrased items were reverse coded in the way that higher scores indicated higher self-concepts. The reliability of the scale according to Geldhof et al. (2014) was high at the within-level (α = 0.92) and the between-level (α = 0.97). Moreover, invariance tests provided evidence for the invariance of the scale across gender groups (see Table 1).

**TABLE 1:** Measurement invariance of math self-concept between gender groups.

Model	Equality constraints	χ^2^	*df*	*p*	CFI	TLI	RMSEA
1	Baseline	179.54	18	<0.001	0.996	0.993	0.113
2	Thresholds	183.46	36	<0.001	0.996	0.997	0.076
3	Thresholds and loadings	161.19	41	<0.001	0.997	0.998	0.065

*The table shows the pooled results over *m* = 50 imputed data sets. They stem from single-level analyses in MPlus in which the complex modeling procedure (*type* = *complex*) was used to correct the estimated standard errors for the nested data structure of students in classes. The self-concept items were treated as categorical indicators and measurement invariance was tested according to Svetina et al. (2020). In Model 1, the indicators were allocated to one factor, but the thresholds and loadings were estimated freely across the groups. In Model 2, the thresholds were constrained to be equal across the groups. In Model 3, the thresholds and loadings were constrained to be equal across the groups. Measurement invariance is supported since the model fit improves with increasing equality constraints. CFI, comparative fit index; TLI, Tucker-Lewis index; RMSEA, root mean square error of approximation. *N* = 1,424 (*n* = 800 girls, *n* = 607 boys, *n* = 17 not specified).*

#### Gender Stereotypes

Students’ beliefs in the stereotype endorsing that math would be a typically male subject were measured using three items: (1) “Boys are simply more gifted at math,” (2) “If there was a typical boy subject, math would be one,” (3) “Math is a subject that is usually more fun for boys.” These items had been developed by Pohlmann (2005). The students responded to all items on a 6-point Likert scale ranging from 1 = *strongly disagree* to 6 = *strongly agree*. The reliability of the scale (Geldhof et al., 2014) was high at the within-level (α = 0.90) and the between-level (α = 0.99).

#### Grades

The students reported their math grades from their latest report cards. Previous research has shown that self-reports of grades in the German school system are sufficiently reliable (e.g., Dickhäuser and Plenter, 2005). All grades were coded according to the German 15-point grading system, which ranges from 0 = *insufficient* to 15 = *perfect*. This grading system is mainly used at the upper secondary level in Germany, which was also the case in the present study. When teachers assigned grades according to the 6-point grading system, ranging from 1 = *excellent* to 6 = *insufficient* (as the case in most of the lower classes in the sample of the present study), I converted these grades into the 15-point system, using the official transformation key, according to which grade 1 equals 14 points, grade 2 equals 11 points, grade 3 equals 8 points, grade 4 equals 5 points, grade 5 equals 2 points, and grade 6 equals 0 points. I preferred this transformation as it did not lead to any loss of information. Nevertheless, I note that the findings of this study fully replicated if transforming the grades from the 15-point system to the 6-point system instead (i.e., 13−15 points to grade 1, 10−12 points to grade 2, etc.).

### Statistical Analyses

The statistical analyses were similar to Muntoni et al. (2021). I conducted multilevel analyses in MPlus 8.4 (Muthén and Muthén, 2017), with students (within-level) being clustered in classes (between-level). More specifically, I specified doubly latent multiple group two-level structural equation models to examine whether students’ math self-concepts were affected by their individual math-related gender stereotypes and the average math-related gender stereotypes within their classes. In these models, students’ gender stereotypes were estimated as latent variables at the within- and between-level, which allowed controlling for measurement and sampling errors (Marsh et al., 2009). Since group membership (i.e., gender) was included as a within-level grouping variable (because each class consisted of girls and boys), the between-level data were not independent between the within-level groups. To account for this dependency, I applied multilevel mixture models, in which a latent class variable indicating the group membership was specified (Asparouhov and Muthén, 2012). Thus, a latent class could be set up at the within-level and the group membership variable was specified as a perfect indicator of the latent class variable (Muntoni et al., 2021). To handle missing values (on average, 2.2% of the data were missing per variable), I applied multiple imputation, including *m* = 50 imputed data sets (Graham et al., 2007).

Overall, I calculated two models. In Model 1, I only took students’ gender stereotypes (independent variable) and self-concepts (dependent variable) into consideration. In Model 2, I additionally controlled for students’ math grades and age (control variables). In both models, I also included 10 dummy variables, indicating students’ affiliation to 1 of 11 schools, which allowed me to take account of the three-level data structure. For testing Hypotheses 1a and 1b, I examined whether the effects of students’ gender stereotypes on their math self-concepts at the within-level were significantly negative for girls and significantly positive for boys. For testing Hypothesis 2, I examined whether contextual effects of class-average gender stereotypes on students’ math self-concepts occurred. To this end, I calculated additional parameters indicating whether the effects of gender stereotypes on self-concepts at the between- and within-level were significantly different from each other. This was necessary because the variables which appeared at both levels were implicitly group-mean centered. Accordingly, the effects of class-average gender stereotypes on math self-concepts at the between-level were no direct estimates of the contextual effects (Marsh et al., 2009). As suggested in Hypothesis 2, the contextual effects should be significantly negative for girls.

I calculated Tymms’ (2004) Δ to facilitate the interpretation of the effect sizes. For this purpose, I used the formula Δ = (2 × *B* × *SD*_*predictor*_)/σ, where *B* is the unstandardized regression coefficient, *SD*_*predictor*_ is the standard deviation of the predictor variable (for the contextual effect: at the between-level), and σ is the total standard deviation of the outcome variable (Marsh et al., 2009). Tymms’ Δ can be interpreted similar to Cohen’s (1988) *d*. Thus, |Δ| = 0.2 represents a small effect, |Δ| = 0.5 a moderate effect, and |Δ| = 0.8 a large effect.

## Results

### Preliminary Analyses

Table 2 presents the descriptive statistics. In line with previous research, boys showed higher math self-concepts compared to girls (Δ*M* = 0.43, β = 0.16, *p* < 0.001), whereas boys’ and girls’ math grades did not significantly differ from each other (Δ*M* = −0.07, β = −0.01, *p* = 0.71). Boys also held a slightly stronger belief in the stereotype favoring boys in math (Δ*M* = 0.19, β = 0.08, *p* = 0.02). Moreover, boys’ math-related gender stereotypes were positively related to their math self-concepts (*r* = 0.18, *p* < 0.001) and math grades (*r* = 0.12, *p* < 0.01), whereas girls’ math-related gender stereotypes were negatively related to their math self-concepts (*r* = −0.26, *p* < 0.001) and math grades (*r* = −0.21, *p* < 0.001). For both genders, older students showed lower math grades (both *r* = −0.14, *p* < 0.01). In addition, older girls showed slightly stronger beliefs in the stereotype favoring boys in math compared to younger girls (*r* = 0.13, *p* < 0.01) and older boys showed slightly lower math self-concepts compared to younger boys (*r* = −0.15, *p* < 0.01). The correlations between students’ math self-concepts and math grades were strongly positive for girls (*r* = 0.61, *p* < 0.001) and boys (*r* = 0.58, *p* < 0.001). The intraclass correlation coefficients (ICCs), indicating the proportion of total variance that was accounted for by between-class variance, were 0.05 or higher for all four variables. In particular, the ICC of 0.11 found for students’ beliefs in the stereotype favoring boys in math is noteworthy, as it indicated that students of the same class showed a substantial agreement in their belief in this stereotype. Furthermore, this value suggested that it was appropriate to examine the relations between students’ math-related gender stereotypes and their math self-concepts using multilevel analyses.

**TABLE 2:** Means, standard deviations, bivariate correlations, and intraclass correlations [with 95% confidence intervals] of the manifest variables.

Variables	*M*_*girls*_	*M*_*boys*_	*SD*_*girls*_	*SD*_*boys*_	*r*_*math self–concept*_	*r*_*gender stereotype*_	*r*_*math grade*_	*r*_*age*_	ICC
Math self-concept	**3.85 [3.72, 3.97]**	**4.27 [4.14, 4.40]**	**1.38 [1.33, 1.43]**	**1.28 [1.21, 1.34]**	−	**0.18 [0.10, 0.27]**	**0.58 [0.52, 0.64]**	**−0.15 [−0.24, −0.06]**	**0.05 [0.02, 0.08]**
Gender stereotype	**2.73 [2.58, 2.87]**	**2.92 [2.78, 3.06]**	**1.25 [1.20, 1.31]**	**1.27 [1.21, 1.33]**	**−0.26 [−0.34, −0.19]**	–	**0.12 [0.04, 0.21]**	0.08 [−0.02,0.18]	**0.11 [0.07, 0.14]**
Math grade	**8.43 [8.12, 8.74]**	**8.36 [8.05, 8.67]**	**3.03 [2.87, 3.18]**	**3.10 [2.87, 3.30]**	**0.61 [0.56, 0.66]**	**−0.21 [−0.29, −0.13]**	–	**−0.14 [−0.23, −0.05]**	**0.09 [0.05, 0.12]**
Age	**15.21 [14.76, 15.66]**	**14.95 [14.52, 15.37]**	**2.05 [1.80, 2.26]**	**1.94 [1.70, 2.15]**	−0.09 [−0.18, 0.01]	**0.13 [0.04, 0.23]**	**−0.14 [−0.23, −0.05]**	–	**0.90 [0.87, 0.92]**

*The table shows the pooled results over *m* = 50 imputed data sets. The means, standard deviations, and bivariate correlations stem from a single-level analysis in MPlus in which the complex modeling procedure (*type* = *complex*) was used to correct the estimated standard errors for the nested data structure of students in classes. The correlations below the diagonal refer to the subsample of girls. The correlations above the diagonal refer to the subsample of boys. The intraclass correlation coefficients (ICCs) indicate the proportion of total variance that is accounted for by between-class variance. They stem from additional multilevel analyses. Math self-concept and gender stereotype had a possible range of 1 = *strongly disagree* to 6 = *strongly agree*. Math grade had a possible range of 0 = *insufficient* to 15 = *perfect*. Bold values are significantly different from zero (*p* < 0.05). *N* = 1,424 (*n* = 800 girls, *n* = 607 boys, *n* = 17 not specified).*

### Hypotheses Testing

Table 3 presents the results of the multilevel analyses. Model 1 tested the effects of students’ math-related gender stereotypes on their math self-concepts without considering control variables. As predicted in Hypothesis 1, girls’ individual math-related gender stereotypes showed a negative effect on their math self-concepts (*B* = −0.26, *p* < 0.001, Δ = −0.43), whereas this effect was positive for boys (*B* = 0.21, *p* < 0.001, Δ = 0.35). Moreover, as predicted in Hypothesis 2, there was a negative contextual effect on girls’ math self-concepts (*B* = −0.38, *p* ≤ 0.05, Δ = −0.23), implying that classmates’ math-related gender stereotypes were negatively related to girls’ math self-concepts, after controlling for individual differences in gender stereotypes. The contextual effect on boys’ math self-concepts was non-significant (*B* = 0.07, *p* = 0.78). Model 2 demonstrated that these relations also persisted after controlling for students’ math grades and age. Whereas the negative effect of girls’ individual math-related gender stereotypes on their math self-concepts (*B* = −0.15, *p* < 0.001, Δ = −0.24) and the positive effect of boys’ individual math-related gender stereotypes on their math self-concepts (*B* = 0.13, *p* < 0.001, Δ = 0.24) were slightly reduced, the negative contextual effect on girls’ math self-concepts was even slightly stronger (*B* = −0.44, *p* = 0.03, Δ = −0.26). The contextual effect on boys’ math self-concepts was still non-significant (*B* = −0.16, *p* = 0.53). Students’ individual math grades showed strong positive effects on their math self-concepts (girls: *B* = 0.28, *p* < 0.001, Δ = 1.19; boys: *B* = 0.24, *p* < 0.001, Δ = 1.12). The effects of math grades at the between-level and of age at both levels were non-significant (all |*B|* ≤ 0.09, *p* ≥ 0.07).

**TABLE 3:** Unstandardized regression coefficients, standard deviations, and Tymms’ Δ [with 95% confidence intervals] of the multilevel analyses predicting students’ math self-concept.

	Model 1						Model 2					
	Girls			Boys			Girls			Boys		
Variables	*B*	*SD*	Δ	*B*	*SD*	Δ	*B*	*SD*	Δ	*B*	*SD*	Δ
**Within-level**												
Gender stereotype	**−0.26 [−0.35, −0.16]**	**1.13 [1.06, 1.19]**	**−0.43 [−0.59, −0.27]**	**0.21 [0.11, 0.30]**	**1.13 [1.06, 1.19]**	**0.35 [0.19, 0.51]**	**−0.15 [−0.22, −0.07]**	**1.13 [1.06, 1.19]**	**−0.24 [−0.37, −0.11]**	**0.13 [0.06, 0.21]**	**1.13 [1.06, 1.19]**	**0.24 [0.10, 0.37]**
Math grade	–	–	–	–	–	–	**0.28 [0.25, 0.31]**	**2.93 [2.79, 3.06]**	**1.19 [1.07, 1.31]**	**0.24 [0.21, 0.27]**	**2.93 [2.79, 3.06]**	**1.12 [0.98, 1.26]**
Age	–	–	–	–	–	–	0.09 [−0.01, 0.19]	**0.65 [0.56, 0.73]**	0.09 [−0.01, 0.18]	−0.06 [−0.19, 0.08]	**0.65 [0.56, 0.73]**	−0.06 [−0.19, 0.08]
**Between-level**												
Gender stereotype	**−0.63 [−0.99, −0.28]**	**0.40 [0.33, 0.47]**	**−0.38 [−0.59, −0.17]**	0.28 [−0.22, 0.79]	**0.40 [0.33, 0.47]**	0.17 [−0.14, 0.48]	**−0.58 [−0.95, −0.21]**	**0.40 [0.33, 0.46]**	**−0.34 [−0.56, −0.13]**	−0.03 [−0.52, 0.46]	**0.40 [0.33, 0.46]**	−0.02 [−0.33, 0.29]
Math grade	–	–	–	–	–	–	0.08 [−0.06, 0.22]	**0.90 [0.67, 1.08]**	0.11 [−0.08, 0.29]	−0.04 [−0.27, 0.19]	**0.90 [0.67, 1.08]**	−0.06 [−0.39, 0.27]
Age	–	–	–	–	–	–	−0.05 [−0.10, 0.01]	**1.91 [1.70, 2.10]**	−0.13 [−0.29, 0.03]	−0.07 [−0.16, 0.03]	**1.91 [1.70, 2.10]**	−0.20 [−0.48, 0.08]
**Contextual effect**												
Gender stereotype	**−0.38 [−0.76, −0.00]**	**0.40 [0.33, 0.47]**	**−0.23 [−0.46, −0.00]**	0.07 [−0.44, 0.59]	**0.40 [0.33, 0.47]**	0.05 [−0.27, 0.36]	**−0.44 [−0.82, −0.05]**	**0.40 [0.33, 0.46]**	**−0.26 [−0.48, −0.03]**	−0.16 [−0.67, 0.34]	**0.40 [0.33, 0.46]**	−0.10 [−0.42, 0.22]

*The table shows the pooled results over *m* = 50 imputed data sets. Tymms’ (2004) Δ was calculated using the formula Δ = (2 × *B* × *SD*_*predictor*_)/σ, where *B* is the unstandardized regression coefficient, *SD*_*predictor*_ is the standard deviation of the predictor variable (for the contextual effect: at the between-level), and σ is the total standard deviation of math self-concept (Model 1: σ = 1.35 for girls, σ = 1.32 for boys; Model 2: σ = 1.37 for girls, σ = 1.27 for boys). Both models also included 10 dummy variables at the between-level, indicating students’ affiliation to 1 of 11 schools (not depicted). Bold values are significantly different from zero (*p* < 0.05). *N* = 1,424 (*n* = 800 girls, *n* = 607 boys, *n* = 17 not specified).*

## Discussion

The present research substantially extends our knowledge of the relations between subject-specific gender stereotypes and self-concepts. For the first time, I investigated how students’ individual and their classmates’ math-related gender stereotypes affect girls’ and boys’ math self-concepts. In line with my hypotheses, and with findings from previous research (e.g., Nosek et al., 2002; Steffens and Jelenec, 2011; Passolunghi et al., 2014), I found that girls showed lower math self-concepts, the more they believed in the stereotype favoring boys in math, whereas boys showed higher math self-concepts, the more they believed in this stereotype. Furthermore, and most central for this research, I found a negative contextual effect of classmates’ beliefs in the stereotype favoring boys in math on girls’ math self-concepts. Thus, girls attending classes in which students strongly believed in the stereotype that math would be a typically male domain showed lower math self-concepts than girls attending classes in which the students did not believe in this stereotype, after controlling for individual gender stereotypes. The contextual effect of classmates’ beliefs in the stereotype favoring boys in math on boys’ math self-concepts was close to zero and indicated that classmates’ shared beliefs in this stereotype do not significantly affect boys’ math self-concepts.

### Theoretical Implications

The findings of this research have important theoretical implications as they further support the role of classmates as significant socializing peers. Specifically, it was shown that girls’ math self-concepts were lower the more their classmates believed in the stereotype favoring boys in math. In contrast, boys’ math self-concepts were not related to the gender stereotypes shared in students’ classrooms. Remarkably, both the significant contextual effect on girls’ math self-concepts and the non-significant contextual effect on boys’ math self-concepts found in the present research were in accord with the results of Muntoni et al. (2021), who found a negative contextual effect of shared reading-related gender stereotypes, endorsing that reading would be a typically female domain, on boys’ reading self-concepts, but not on girls’ reading self-concepts. Taken together the results of both studies, it seems that gender stereotypes shared in the classroom negatively affect the self-concepts of students of the stereotyped gender group, whereas they do not affect the self-concepts of students of the non-stereotyped gender group.

Muntoni et al. (2021) had already speculated, when they compared the effects of gender stereotypes between the domains of math and reading, “that the underlying processes are rather similar” (p. 190). Nevertheless, an empirical test of this assumption seemed indicated, especially because math-related gender stereotypes are even less tenable than reading-related gender stereotypes, according to differences in girls’ and boys’ performance in standardized math and reading tests: Whereas girls, in fact, do not perform significantly worse in math than boys (which also corresponds to the similar math grades found for girls and boys in the present study), they still outperform boys in reading [e.g., Organisation for Economic Co-operation and Development (OECD), 2020]. It would, therefore, also have been plausible if girls’ math self-concepts had not been related to shared math-related gender stereotypes in the present study, although Muntoni et al. (2021) found a negative effect of shared reading-related gender stereotypes on boys’ reading self-concepts.

However, the findings of the present study suggest that the impact of math-related gender stereotypes on girls’ math self-concepts may be even stronger than the impact of reading-related gender stereotypes on boys’ reading self-concepts. This is due to the fact that the contextual effect on girls’ math self-concepts occurred beyond the negative effect of girls’ individual math-related gender stereotypes on their math self-concepts, whereas the contextual effect on boys’ reading self-concepts in Muntoni et al.’s (2021) study, which was similarly strong as the contextual effect on girls’ math self-concepts in the present study, occurred along with a non-significant effect of boys’ individual reading-related gender stereotypes on their reading self-concepts. More precisely, Muntoni et al. (2021) examined two models. In the first model, they only controlled for demographics. In this model, they found positive effects of girls’ individual reading-related gender stereotypes on their reading self-concepts and negative effects of boys’ individual reading-related gender stereotypes on their reading self-concepts, but no contextual effects. The contextual effect on boys’ reading self-concepts was only shown in the second model, in which the authors additionally controlled for prior reading self-concepts. Yet, in this model, the effects of gender stereotypes at the within-level were non-significant. The fact that the present research revealed simultaneous effects of students’ individual and shared gender stereotypes on their self-concepts constitutes an important difference to Muntoni et al.’s (2021) results. Although the results of both studies suggest that shared gender stereotypes have specific effects on the self-concepts of the stereotyped group, it seems that there are also some differences in the mechanisms of how gender stereotypes affect students’ self-concepts in the domains of math and reading.

### Practical Implications

The findings of this research are of high practical importance as they illustrate, more differentiated than in previous research, how girls’ math self-concepts are impaired by math-related gender stereotypes. On the one hand, it was demonstrated that girls show lower math self-concepts, the more they believe in the stereotype favoring boys in math. This finding was already known from prior studies (e.g., Passolunghi et al., 2014). However, in the present study, it was shown for the first time that these relations also hold in multilevel analyses. On the other hand, the present research revealed that math-related gender stereotypes shared in the classroom negatively relate to girls’ math self-concepts, after controlling for girls’ individual math-related gender stereotypes. To the best of my knowledge, this finding has not been shown in any prior empirical study yet. To conclude, the math-related gender stereotype endorsing that math would be a typically male domain is not only incompatible with empirical findings that showed no substantial differences in girls’ and boys’ math performance [e.g., Organisation for Economic Co-operation and Development (OECD), 2020]. It also seems to be disadvantageous for girls in two ways. Teachers are, therefore, advised to avoid the emergence of gender stereotypes and to remove existing stereotypes whenever possible. In particular, this applies if gender stereotypes manifest themselves in the classroom and thus affect students’ self-concepts (see, e.g., Frawley, 2005, for interventional strategies).

It is worth noting that the effects of students’ individual and shared math-related gender stereotypes found in the present study were only small—similarly to the effects of students’ individual and shared reading-related gender stereotypes found by Muntoni et al. (2021). Nevertheless, it seems likely that these effects will have a substantial impact over time and could even become a self-fulfilling prophecy. Several studies have demonstrated that students’ academic self-concepts are not only formed based on their prior achievements but also affect their subsequent achievements to a substantial degree (e.g., Marsh and Craven, 2006; Wolff et al., 2020b). If girls’ math self-concepts suffer from math-related stereotypes, this can consequently impair their future math performance. In particular, girls might then be prone to believe that they indeed perform worse in math than boys, with the result that their math self-concepts and achievements could further worsen—and that they might decide against a career in math, science, technology, or engineering, even though they have the potential to succeed in these disciplines.

### Strengths, Limitations, and Directions for Future Research

The present research has some limitations that should be discussed in greater depth. A first limitation is its cross-sectional design. Accordingly, the findings of this study allow us to make conclusions about how students’ math-related gender stereotypes are related to their math self-concepts measured at a certain point in time, but not to changes in these self-concepts across time. Future research should, therefore, aim to examine how individual and shared math-related gender stereotypes relate to changes in students’ self-concepts. In particular, it would seem worthwhile to conduct such investigations during phases of school transitions, which have shown to come along with significant changes in students’ self-concepts (e.g., Wigfield et al., 1991; Wolff et al., 2020c), given that these changes might also result from changes in gender stereotypes shared within the classroom. Moreover, it would be advisable for future research to measure students’ gender stereotypes (and other covariates) some time before the self-concepts as such a design would be more suitable to allow an approximation to causal conclusions than the simultaneous assessment of these constructs.

A second limitation of the present study can be seen in the fact that the classes were selected by the school administrators, rather than chosen randomly. Accordingly, the examined sample was not representative. However, this limitation seemed acceptable since the aim of this research was not to examine gender stereotypes and self-concepts in a representative student sample, but the relations between these constructs at the individual and class level. For this purpose, the analyzed data seemed quite suitable as they showed several favorable characteristics. For example, the internal consistencies of the self-concept scale and the scale assessing students’ gender stereotypes were very high, the ICC of students’ gender stereotypes was also high, and the number of missing values was very low. Beyond that, the data were collected in the year 2018, specifically for the present study. Given that gender stereotypes have changed across the decades (e.g., Eagly et al., 2020), this up-to-datedness of the analyzed data can be seen as a strength of this study.

A third limitation of the present research is its restriction to the investigation of the relations between students’ subject-specific gender stereotypes and self-concepts in the domain of math. For this reason, it was not possible to test whether Muntoni et al.’s (2021) findings, which referred to these relations in the domain of reading, would have replicated within the present sample. Moreover, it was not possible to examine the interplay between gender stereotypes and self-concepts in different subjects. For example, numerous studies examining the relations between students’ math and verbal achievements and self-concepts have demonstrated that students’ achievement in one domain negatively affects their self-concept in the other domain, after controlling for achievement in the other domain (Möller et al., 2020). It would be conceivable that similar results also emerged for the relations between math and verbal gender stereotypes and self-concepts. Hence, future research should investigate the relations between math- and reading-related gender stereotypes and self-concepts within the same sample.

Finally, a limitation of the present research involves the fact that students’ stereotypes were only measured through self-reports. It is conceivable that students’ implicit gender stereotypes (shared in the classroom) affect their self-concepts beyond what is explicitly expressed. This seems particularly plausible considering that previous studies have found some gender-specific differences in the existence of explicit and implicit gender stereotypes, although the results were somewhat inconsistent (e.g., Steffens and Jelenec, 2011; vs. Passolunghi et al., 2014; see also Nosek et al., 2002). Researchers should thus feel encouraged to measure students’ subject-specific gender stereotypes using both explicit and implicit measures in future research. Furthermore, it would seem worthwhile to supplement explicit self-concept measures with implicit measures of subject-specific self-concepts (e.g., Wolff et al., 2020a), given that gender stereotypes, especially if measured implicitly, might affect students’ self-concepts especially at an unconscious level.

## Conclusion

The present research provided new significant insights into the interaction of gender and gender stereotypes in the formation of students’ academic self-concepts. For the first time, it was shown that not only students’ individual math-related gender stereotypes but also the math-related gender stereotypes shared in their classrooms affect students’ math self-concepts. By analogy with Muntoni et al. (2021), who found a negative contextual effect of classmates’ shared reading-related gender stereotypes on boys’ reading self-concepts, this research revealed a negative contextual effect of classmates’ shared math-related gender stereotypes on girls’ math self-concepts. Taken together, it seems that stereotyped gender groups suffer from both individual and shared gender stereotypes. Given this, it is to be hoped that gender stereotypes favoring girls or boys in a certain domain will continue to decrease in the future.

## Data Availability Statement

The raw data supporting the conclusions of this article will be made available by the author, without undue reservation.

## Ethics Statement

Ethical review and approval was not required for the study on human participants in accordance with the Local Legislation and Institutional Requirements. Written informed consent to participate in this study was provided by the participants’ legal guardian/next of kin.

## Author Contributions

FW designed the study, prepared the research materials, organized the data collection, conducted the analyses, and wrote the manuscript.

## Conflict of Interest

The author declares that the research was conducted in the absence of any commercial or financial relationships that could be construed as a potential conflict of interest.
